# Supplementary material for: Comparison of a Peripheral Nerve Block versus Spinal Anesthesia in Foot or Ankle Surgery: A Systematic Review and Meta-Analysis with a Trial Sequential Analysis
Source: J Pers Med. 2023 Jul 4;13(7):1096. doi: 10.3390/jpm13071096 (PMC10381348; doi:10.3390/jpm13071096)

Supplementary Figure S1. **The trial sequential analysis for the studies comparing the effect of PNB to that of SA on conversion to general anesthesia.** Horizontal line represents the conventional boundaries for statistical significance. The blue solid line represents the cumulative z-curve. The number on the x-axis indicates required information size.

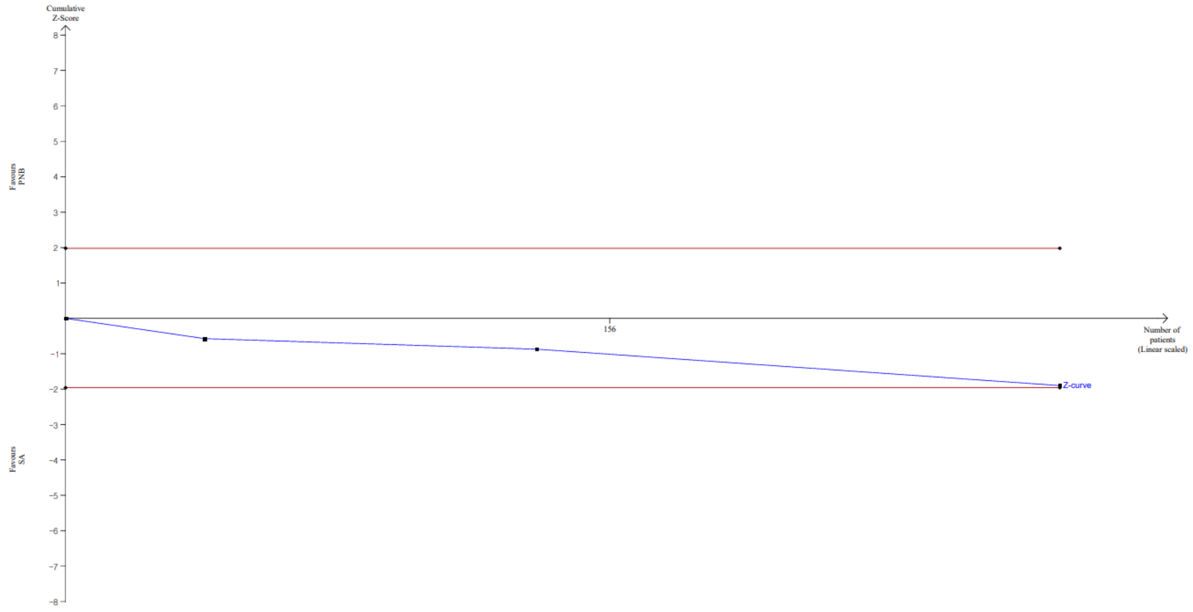

Supplementary Figure S2 Sensitivity analysis excluding one study at a time for the block performance time. The figure depicts individual trials as filled squares with relative sample size and the 95% confidence interval (CI) of the difference as a solid line. The diamond shape indicates the pooled estimate and uncertainty for the combined effect.

Study name	Statistics with study removed		
	Point	Lower limit	Upper limit
Dabir 2020	7.729	5.651	9.807
Karaarslan 2016	7.651	5.753	9.550
Xu 2018	6.545	6.088	7.002
Yang 2016	7.924	6.029	9.818
	7.470	6.072	8.868

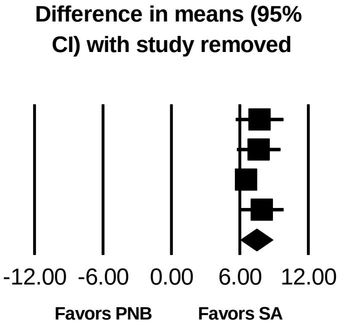

Supplementary Figure S3. The trial sequential analysis for the studies comparing the effect of PNB to that of SA on the block performance time. Uppermost and lowermost curves represent trial sequential monitoring boundary lines for benefit and harm respectively. Horizontal line represents the conventional boundaries for statistical significance. Triangular lines on the right side reflects the futility boundaries. The blue solid line represents the cumulative z-curve. The number on the x-axis indicates required information size.

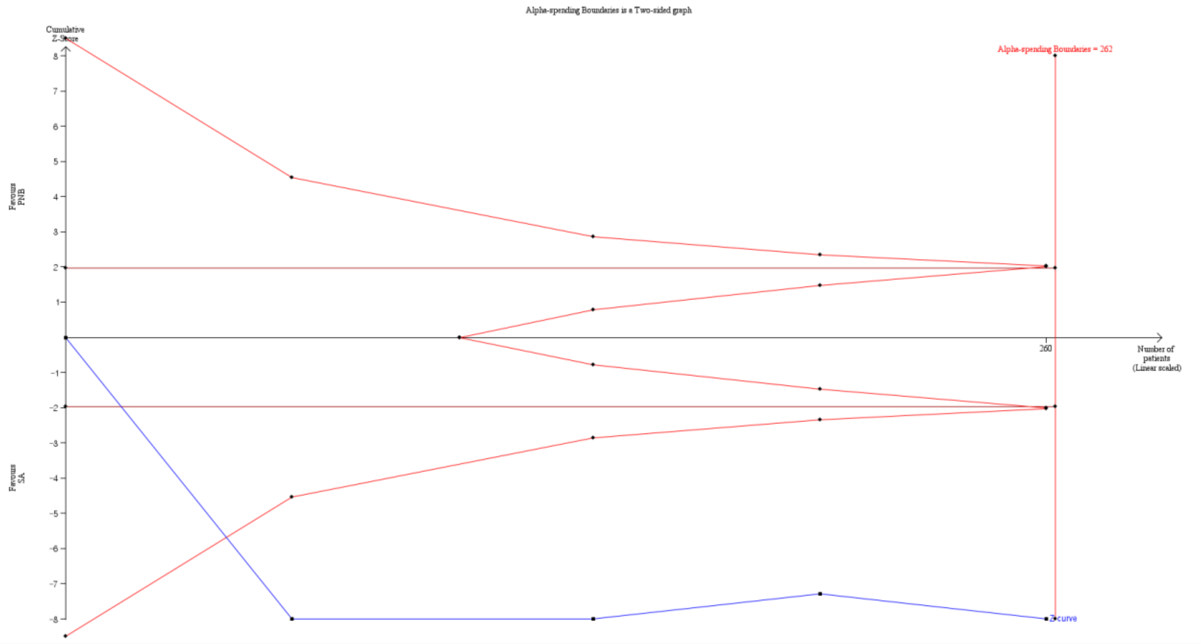

Supplementary Figure S4. Forest plot for studies comparing the effect of PNB to that of SA on the onset time of the sensory block. The figure depicts individual trials as filled squares with relative sample size and the 95% confidence interval (CI) of the difference as a solid line. The diamond shape indicates the pooled estimate and uncertainty for the combined effect.

Study name	Statistics for each study		
	Difference in means	Lower limit	Upper limit
Protic 2010	3.000	1.575	4.425
Xu 2018	3.330	3.044	3.616
Yang 2016	8.900	8.484	9.316
Chauhan2023	14.664	13.774	15.554
	7.483	2.837	12.130

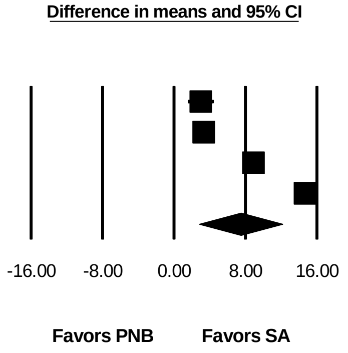

Supplementary Figure S5. Sensitivity analysis excluding one study at a time for the onset time of the sensory block. The figure depicts individual trials as filled squares with relative sample size and the 95% confidence interval (CI) of the difference as a solid line. The diamond shape indicates the pooled estimate and uncertainty for the combined effect.

Study name	Point	Lower limit	Upper limit
Protic 2010	8.950	3.443	14.456
Xu 2018	8.886	3.843	13.930
Yang 2016	7.002	-0.749	14.754
Chauhan2023	5.098	0.783	9.414
	7.483	2.837	12.130

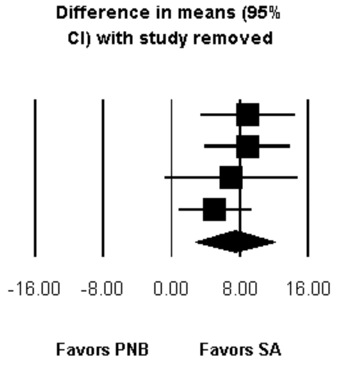

Supplementary Figure S6. The trial sequential analysis for the studies comparing the effect of PNB to that of SA on onset time of the sensory block.

Uppermost and lowermost curves represent trial sequential monitoring boundary lines for benefit and harm respectively. Horizontal line represents the conventional boundaries for statistical significance. Triangular lines on the right side reflects the futility boundaries. The blue solid line represents the cumulative z-curve. The number on the x-axis indicates required information size.

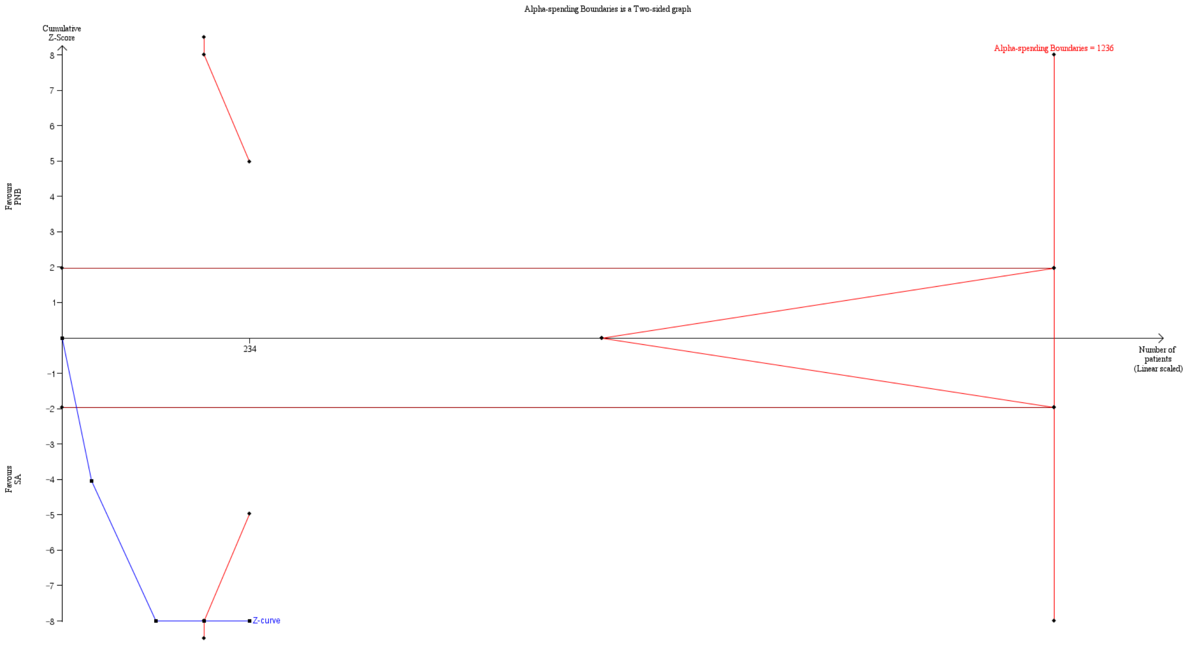

Supplementary Figure S7. Sensitivity analysis excluding one study at a time for the onset time of motor block. The figure depicts individual trials as filled squares with relative sample size and the 95% confidence interval (CI) of the difference as a solid line. The diamond shape indicates the pooled estimate and uncertainty for the combined effect.

Study name	Statistics with study removed		
	Point	Lower limit	Upper limit
Casati 2002	10.190	4.796	15.585
Dabir 2020	10.270	5.369	15.170
Karaarslan 2016	6.458	1.400	11.516
Protic 2010	9.417	3.752	15.082
Xu 2018	10.386	5.052	15.719
Yang 2016	8.978	1.674	16.282
Chauhan2023	7.804	2.781	12.827
	9.071	4.049	14.094

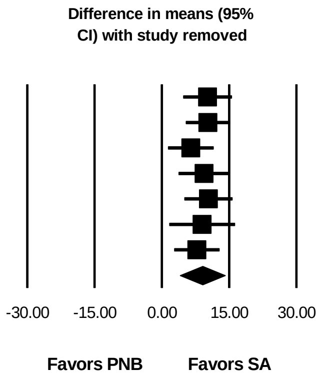

Supplementary Figure S8. The trial sequential analysis for the studies comparing the effect of PNB to that of SA on the onset time of motor block. Uppermost and lowermost curves represent trial sequential monitoring boundary lines for benefit and harm respectively. Horizontal line represents the conventional boundaries for statistical significance. Triangular lines on the right side reflects the futility boundaries. The blue solid line represents the cumulative z-curve. The number on the x-axis indicates required information size.

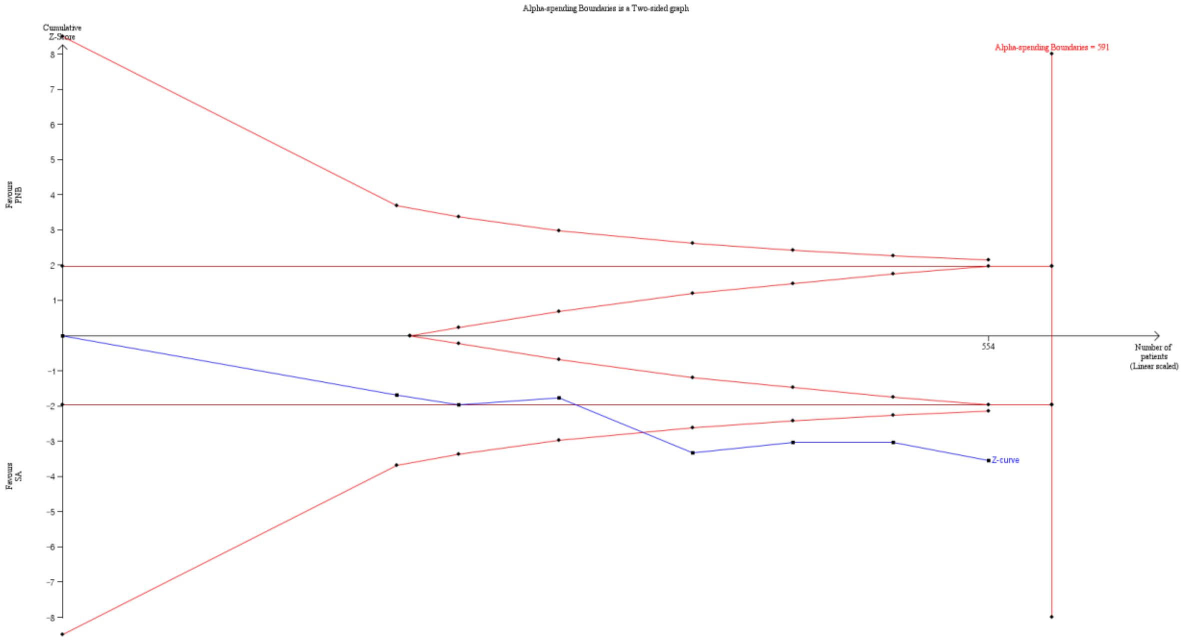

Supplementary Figure S9. Sensitivity analysis excluding one study at a time for the duration of the sensory block. The figure depicts individual trials as filled squares with relative sample size and the 95% confidence interval (CI) of the difference as a solid line. The diamond shape indicates the pooled estimate and uncertainty for the combined effect.

Study name	Statistics with study removed		
	Point	Lower limit	Upper limit
Casati 2002	473.996	321.881	626.112
Lai 2020	481.950	341.574	622.327
Protic 2010	444.986	298.501	591.471
Sort 2021	405.229	357.620	452.839
Xu 2018	463.743	301.764	625.721
Yang 2016	471.690	319.529	623.852
Chauhan2023	467.818	320.908	614.727
	458.530	328.296	588.765

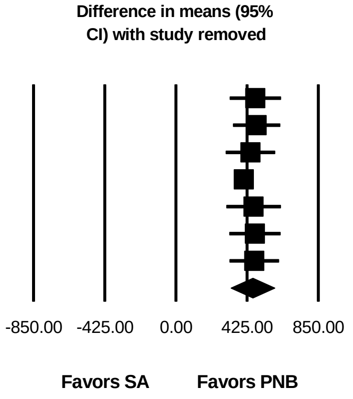

Supplementary Figure S10. The trial sequential analysis for the studies comparing the effect of PNB to that of SA on the duration of the sensory block. Uppermost and lowermost curves represent trial sequential monitoring boundary lines for benefit and harm respectively. Horizontal line represents the conventional boundaries for statistical significance. Triangular lines on the right side reflects the futility boundaries. The blue solid line represents the cumulative z-curve. The number on the x-axis indicates required information size.

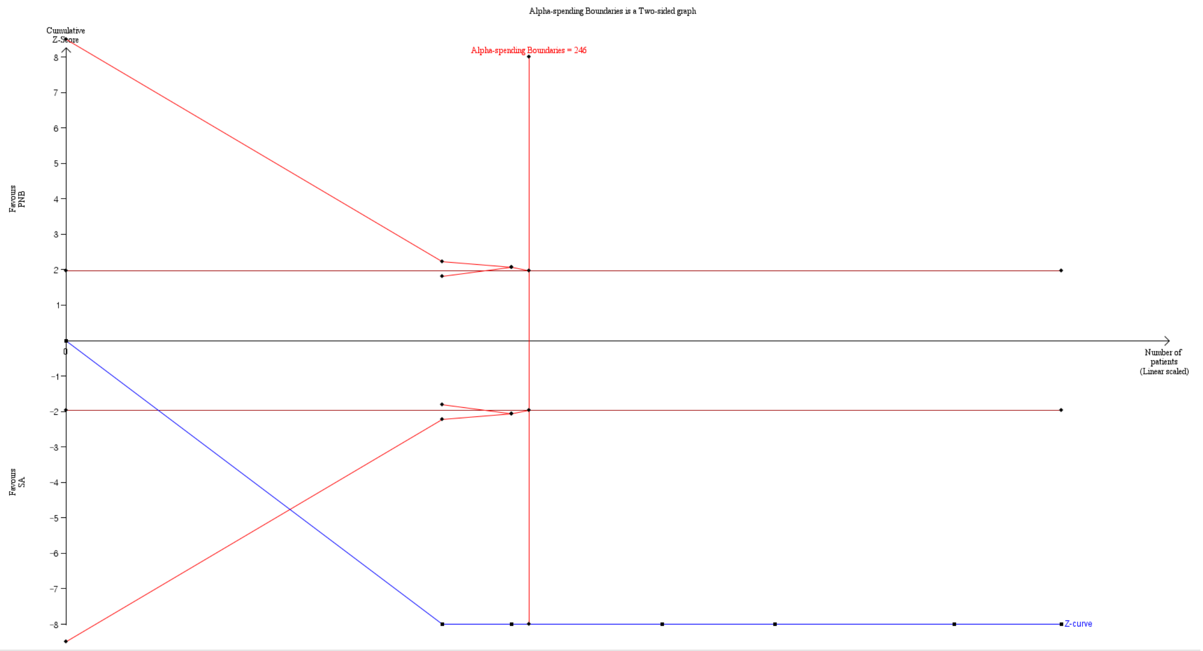

Supplementary Figure S11. Forest plot for studies comparing the effect of PNB to that of SA on the duration of the motor block. The figure depicts individual trials as filled squares with relative sample size and the 95% confidence interval (CI) of the difference as a solid line. The diamond shape indicates the pooled estimate and uncertainty for the combined effect.

Study name	Statistics for each study		
	Difference in means	Lower limit	Upper limit
Casati 2002	154.000	110.106	197.894
Xu 2018	483.820	426.017	541.623
Yang 2016	112.000	67.640	156.360
Chauhan2023	243.333	188.290	298.376
	247.416	95.625	399.208

Difference in means and 95% CI

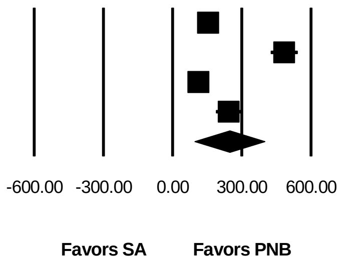

Supplementary Figure S12. Sensitivity analysis excluding one study at a time for the duration of the motor block. The figure depicts individual trials as filled squares with relative sample size and the 95% confidence interval (CI) of the difference as a solid line. The diamond shape indicates the pooled estimate and uncertainty for the combined effect.

Study name	Statistics with study removed		
	Point	Lower limit	Upper limit
Casati 2002	279.125	66.187	492.062
Xu 2018	168.083	97.144	239.021
Yang 2016	293.060	103.043	483.077
Chauhan2023	249.104	41.595	456.613
	247.416	95.625	399.208

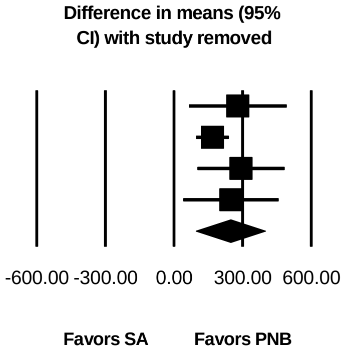

Supplementary Figure S13. Forest plot for studies comparing the effect of PNB to that of SA on the postoperative analgesics requirements. The figure depicts individual trials as filled squares with relative sample size and the 95% confidence interval (CI) of the difference as a solid line. The diamond shape indicates the pooled estimate and uncertainty for the combined effect.

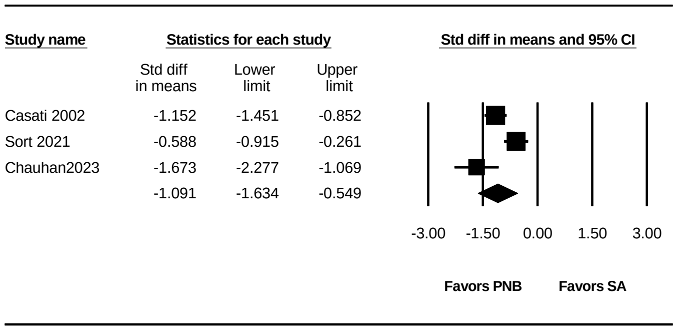

Supplementary Figure S14. Sensitivity analysis excluding one study at a time for the postoperative analgesics requirements. The figure depicts individual trials as filled squares with relative sample size and the 95% confidence interval (CI) of the difference as a solid line. The diamond shape indicates the pooled estimate and uncertainty for the combined effect.

Study name	Point	Lower limit	Upper limit
Casati 2002	-1.099	-2.161	-0.038
Sort 2021	-1.343	-1.836	-0.851
Chauhan2023	-0.874	-1.426	-0.321
	-1.091	-1.634	-0.549

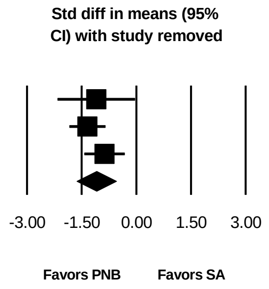

Supplementary Figure S15. The trial sequential analysis for the studies comparing the effect of PNB to that of SA on the postoperative analgesics requirements

. Uppermost and lowermost curves represent trial sequential monitoring boundary lines for benefit and harm respectively. Horizontal line represents the conventional boundaries for statistical significance. Triangular lines on the right side reflects the futility boundaries. The blue solid line represents the cumulative z-curve. The number on the x-axis indicates required information size.

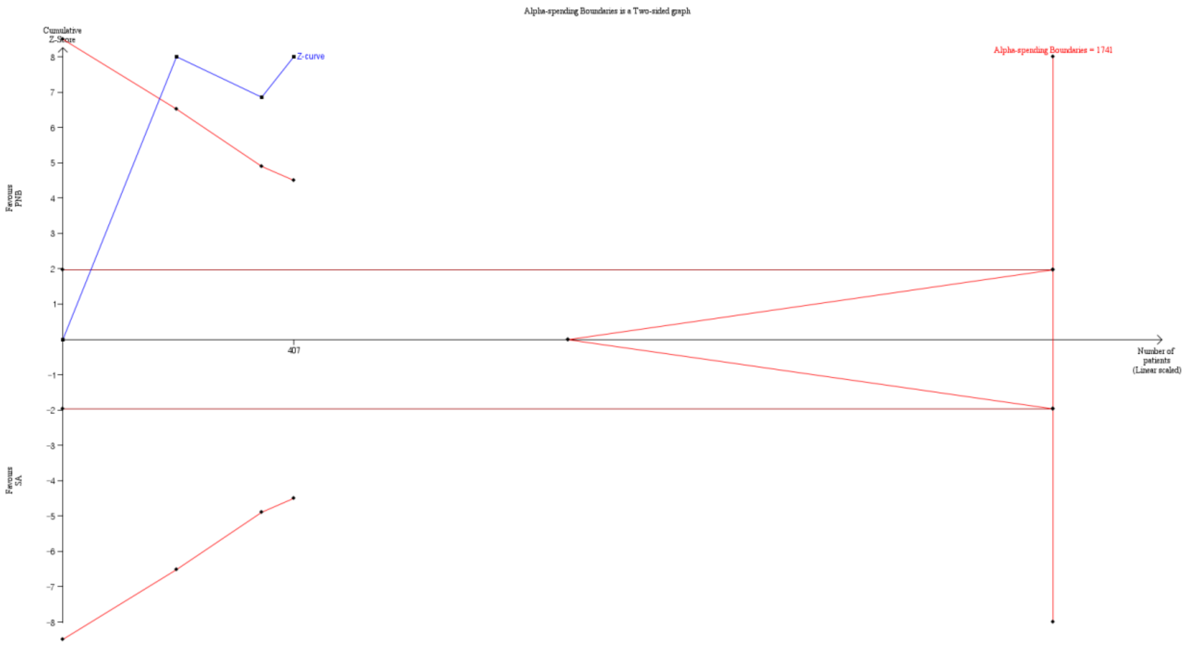

Supplementary Figure S16. Forest plot for studies comparing the effect of PNB to that of SA on the incidence of hypotension. The figure depicts individual trials as filled squares with relative sample size and the 95% confidence interval (CI) of the difference as a solid line. The diamond shape indicates the pooled estimate and uncertainty for the combined effect.

Study name	Statistics for each study		
	Risk ratio	Lower limit	Upper limit
Karaarslan 2016	0.050	0.000	28.081
Lai 2020	0.085	0.012	0.628
Protic 2010	0.352	0.040	3.080
Yang 2016	0.143	0.008	2.679
	0.152	0.042	0.548

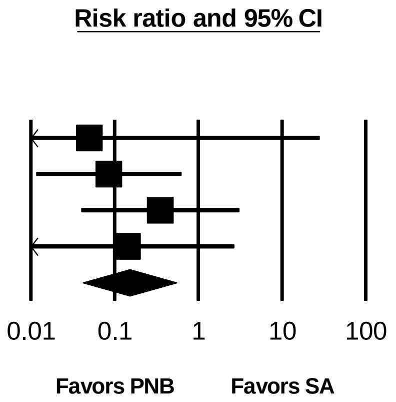

Supplementary Figure S17. The trial sequential analysis for the studies comparing the effect of PNB to that of SA on the incidence of hypotension

. Uppermost and lowermost curves represent trial sequential monitoring boundary lines for benefit and harm respectively. Horizontal line represents the conventional boundaries for statistical significance. Triangular lines on the right side reflects the futility boundaries. The blue solid line represents the cumulative z-curve. The number on the x-axis indicates required information size.

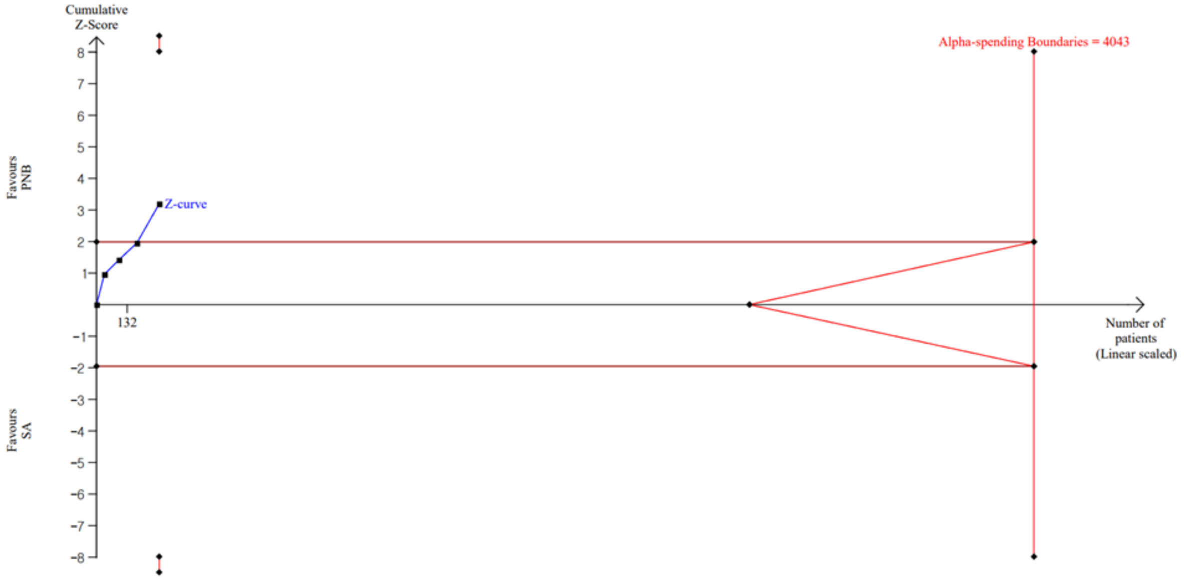

Supplementary Figure S18. Forest plot for studies comparing the effect of PNB to that of SA on the use of vasoactive drug medication. The figure depicts individual trials as filled squares with relative sample size and the 95% confidence interval (CI) of the difference as a solid line. The diamond shape indicates the pooled estimate and uncertainty for the combined effect.

Study name	Statistics for each study		
	Risk ratio	Lower limit	Upper limit
Karaarslan 2016	0.050	0.000	28.081
Lai 2020	0.019	0.000	9.463
Sort 2021	0.279	0.108	0.717
	0.253	0.101	0.638

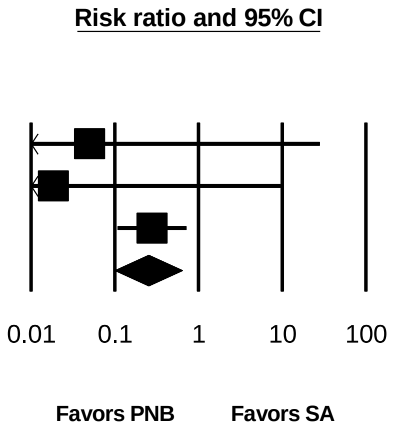

Supplementary Figure S19. The trial sequential analysis for the studies comparing the effect of PNB to that of SA on the use of vasoactive drug medication. Uppermost and lowermost curves represent trial sequential monitoring boundary lines for benefit and harm respectively. Horizontal line represents the conventional boundaries for statistical significance. Triangular lines on the right side reflects the futility boundaries. The blue solid line represents the cumulative z-curve. The number on the x-axis indicates required information size.

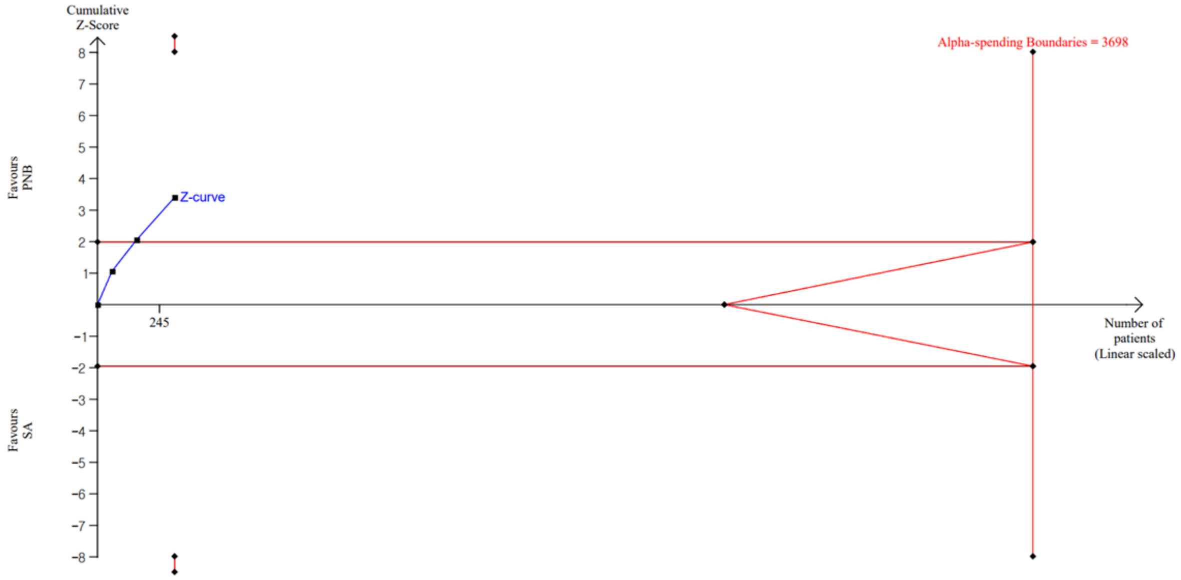

Supplementary Figure S20. Forest plot for studies comparing the effect of PNB to that of SA on the systolic blood pressure at T0. The figure depicts individual trials as filled squares with relative sample size and the 95% confidence interval (CI) of the difference as a solid line. The diamond shape indicates the pooled estimate and uncertainty for the combined effect.

Study name	Statistics for each study		
	Difference in means	Lower limit	Upper limit
Lai 2020	0.255	-9.440	9.950
Xu 2018	-3.800	-9.800	2.200
Chauhan2023	4.652	-3.013	12.317
	-0.112	-5.392	5.168

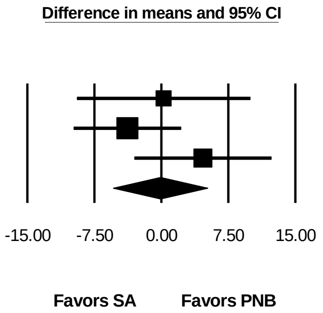

Supplementary Figure S21. Forest plot for studies comparing the effect of PNB to that of SA on the systolic blood pressure at T30. The figure depicts individual trials as filled squares with relative sample size and the 95% confidence interval (CI) of the difference as a solid line. The diamond shape indicates the pooled estimate and uncertainty for the combined effect.

Study name	Statistics for each study		
	Difference in means	Lower limit	Upper limit
Lai 2020	23.279	13.165	33.393
Xu 2018	14.640	8.121	21.159
Chauhan2023	5.052	-2.987	13.091
	13.950	4.603	23.298

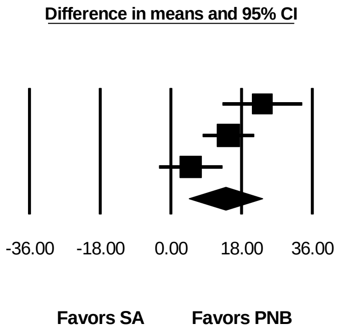

Supplementary Figure S22. Sensitivity analysis excluding one study at a time for the systolic blood pressure at T0. The figure depicts individual trials as filled squares with relative sample size and the 95% confidence interval (CI) of the difference as a solid line. The diamond shape indicates the pooled estimate and uncertainty for the combined effect.

Study name	Point	Lower limit	Upper limit
Lai 2020	10.146	0.769	19.524
Xu 2018	13.896	-3.958	31.751
Chauhan2023	18.059	9.779	26.338
	13.950	4.603	23.298

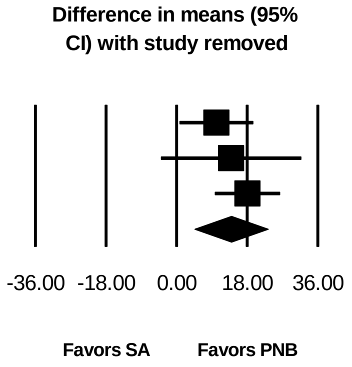

Supplementary Figure S23. The trial sequential analysis for the studies comparing the effect of PNB to that of SA on the systolic blood pressure at T0. Uppermost and lowermost curves represent trial sequential monitoring boundary lines for benefit and harm respectively. Horizontal line represents the conventional boundaries for statistical significance. Triangular lines on the right side reflects the futility boundaries. The blue solid line represents the cumulative z-curve. The number on the x-axis indicates required information size.

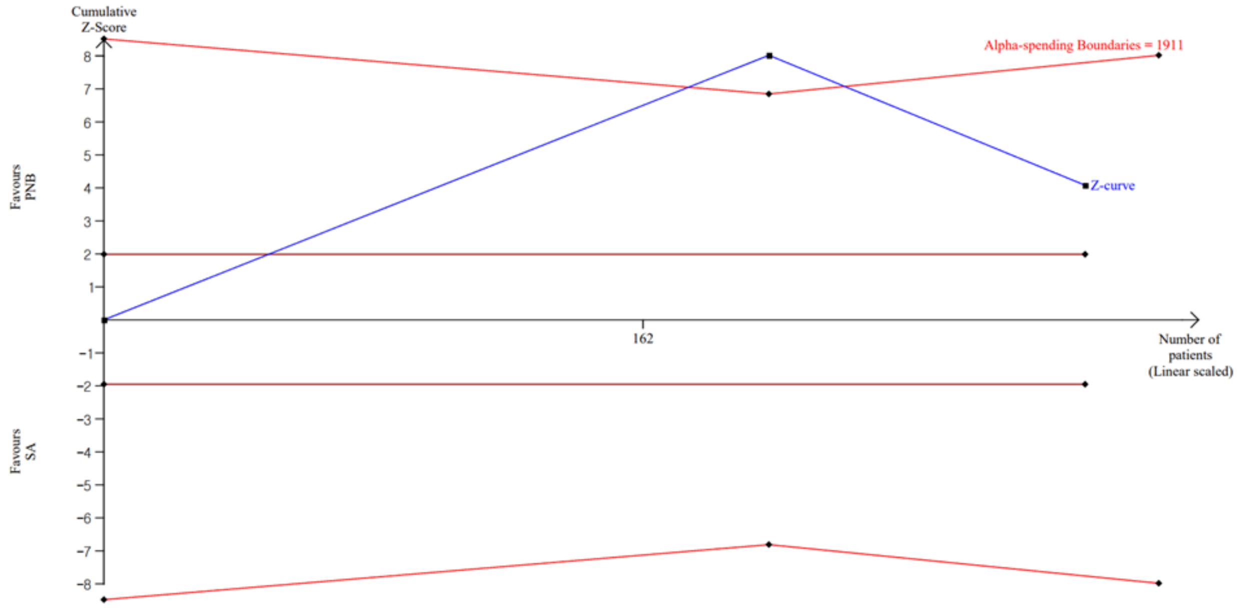

Supplementary Figure S24. The trial sequential analysis for the studies comparing the effect of PNB to that of SA on the systolic blood pressure at T30. Uppermost and lowermost curves represent trial sequential monitoring boundary lines for benefit and harm respectively. Horizontal line represents the conventional boundaries for statistical significance. Triangular lines on the right side reflects the futility boundaries. The blue solid line represents the cumulative z-curve. The number on the x-axis indicates required information size.

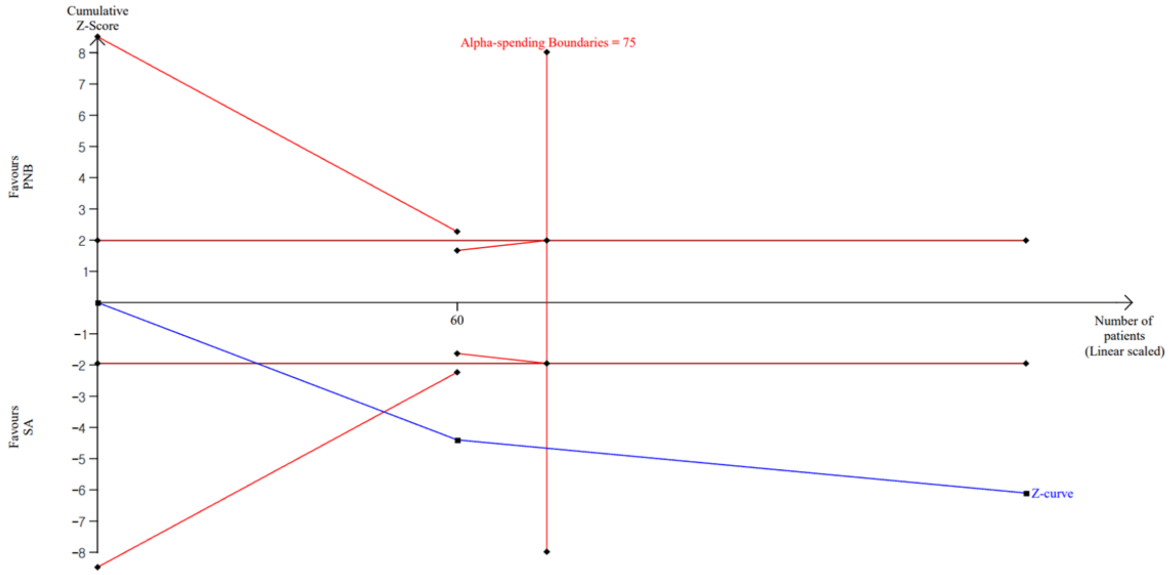

Supplementary Figure S25. Forest plot for studies comparing the effect of PNB to that of SA on the diastolic blood pressure at T0. The figure depicts individual trials as filled squares with relative sample size and the 95% confidence interval (CI) of the difference as a solid line. The diamond shape indicates the pooled estimate and uncertainty for the combined effect.

Study name	Statistics for each study		
	Difference in means	Lower limit	Upper limit
Lai 2020	-2.468	-8.821	3.885
Xu 2018	-1.430	-5.218	2.358
Chauhan 2023	-0.526	-5.014	3.962
	-1.297	-3.931	1.337

Difference in means and 95% CI

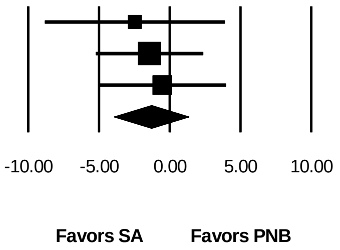

Supplementary Figure S26. Forest plot for studies comparing the effect of PNB to that of SA on the diastolic blood pressure at T30. The figure depicts individual trials as filled squares with relative sample size and the 95% confidence interval (CI) of the difference as a solid line. The diamond shape indicates the pooled estimate and uncertainty for the combined effect.

Study name	Statistics for each study		
	Difference in means	Lower limit	Upper limit
Lai 2020	7.838	1.832	13.843
Xu 2018	6.960	3.134	10.786
Chauhan2023	-0.852	-5.346	3.642
	4.535	-0.969	10.039

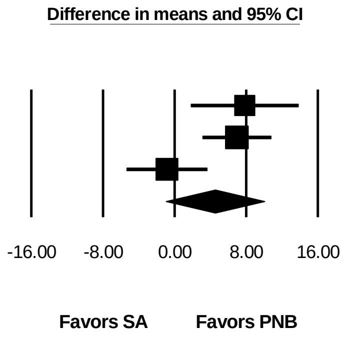

Supplementary Figure S27. The trial sequential analysis for the studies comparing the effect of PNB to that of SA on the diastolic blood pressure at T0. Uppermost and lowermost curves represent trial sequential monitoring boundary lines for benefit and harm respectively. Horizontal line represents the conventional boundaries for statistical significance. Triangular lines on the right side reflects the futility boundaries. The blue solid line represents the cumulative z-curve. The number on the x-axis indicates required information size.

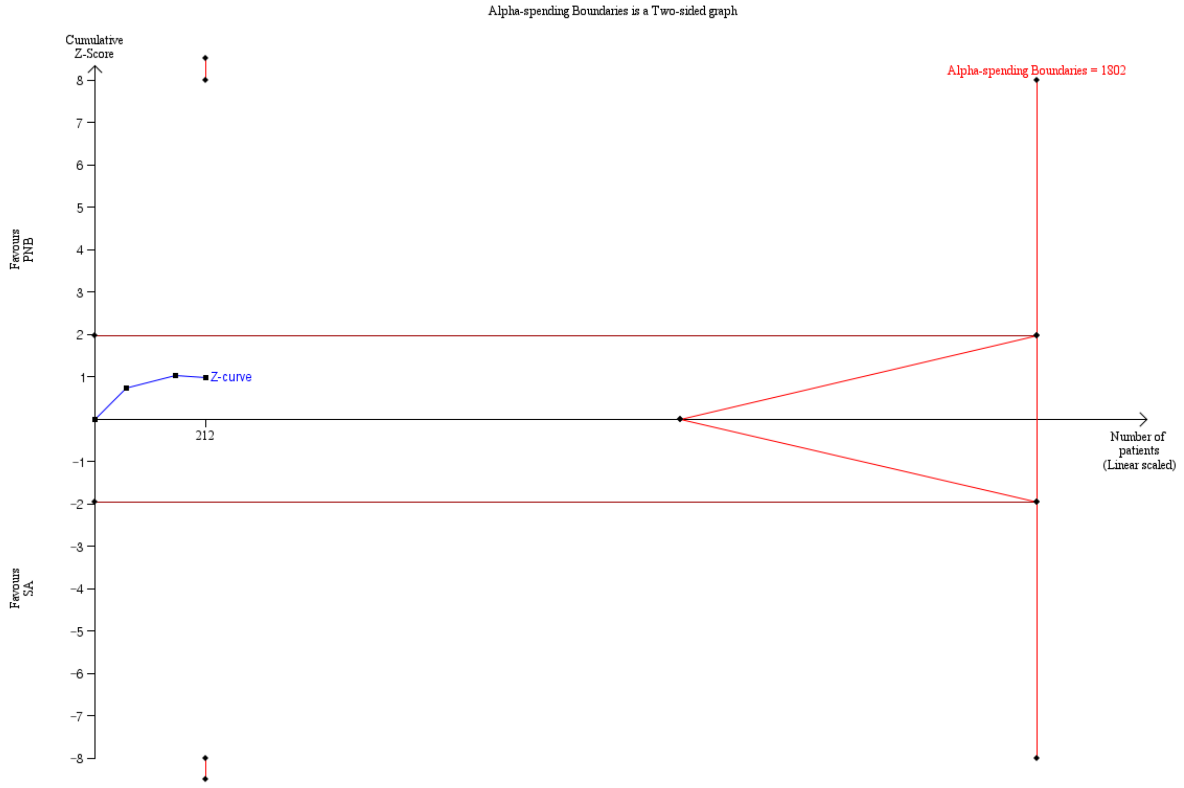

Supplementary Figure S28. The trial sequential analysis for the studies comparing the effect of PNB to that of SA on the diastolic blood pressure at T30. Uppermost and lowermost curves represent trial sequential monitoring boundary lines for benefit and harm respectively. Horizontal line represents the conventional boundaries for statistical significance. Triangular lines on the right side reflects the futility boundaries. The blue solid line represents the cumulative z-curve. The number on the x-axis indicates required information size. The number on the x-axis indicates required information size.

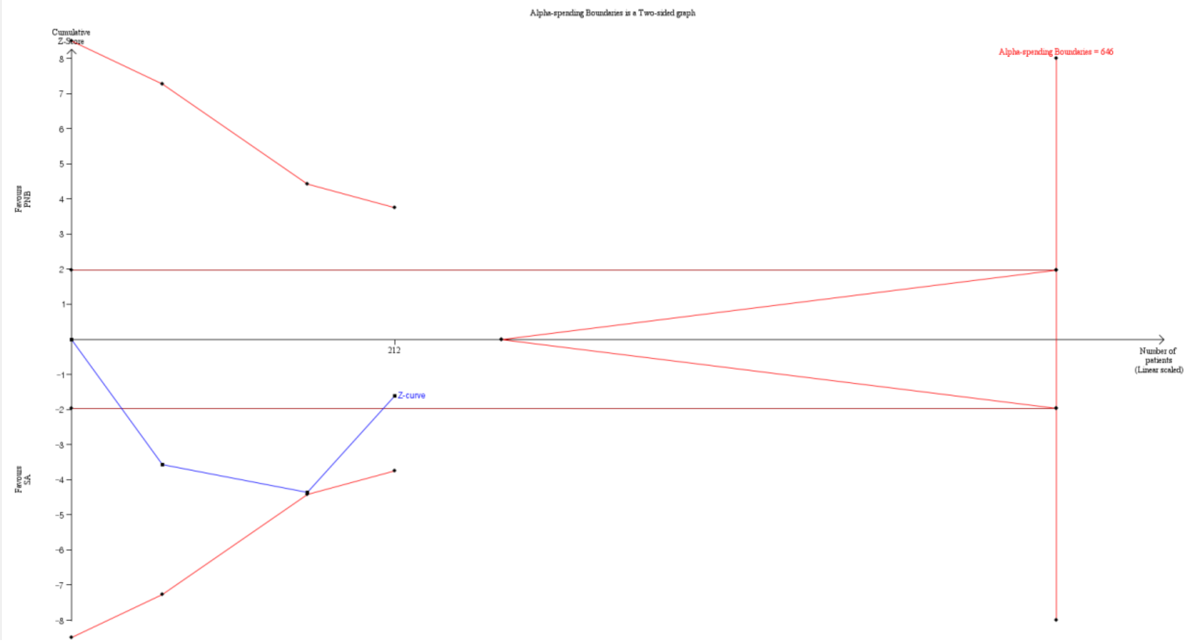

Supplementary Figure S29. Forest plot for studies comparing the effect of PNB to that of SA on the heart rate at T0. The figure depicts individual trials as filled squares with relative sample size and the 95% confidence interval (CI) of the difference as a solid line. The diamond shape indicates the pooled estimate and uncertainty for the combined effect.

Study name	Statistics for each study		
	Difference in means	Lower limit	Upper limit
Lai 2020	-3.140	-8.673	2.393
Xu 2018	0.970	-4.617	6.557
Chauhan2023	3.148	-4.438	10.734
	-0.204	-3.696	3.288

Difference in means and 95% CI

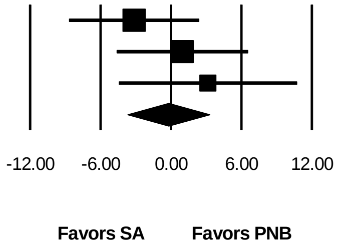

Supplementary Figure S30. Forest plot for studies comparing the effect of PNB to that of SA on the heart rate at T30. The figure depicts individual trials as filled squares with relative sample size and the 95% confidence interval (CI) of the difference as a solid line. The diamond shape indicates the pooled estimate and uncertainty for the combined effect.

Study name	Statistics for each study		
	Difference in means	Lower limit	Upper limit
Lai 2020	7.140	2.122	12.158
Xu 2018	3.360	-1.999	8.719
Chauhan2023	-4.441	-12.288	3.407
	2.671	-3.256	8.599

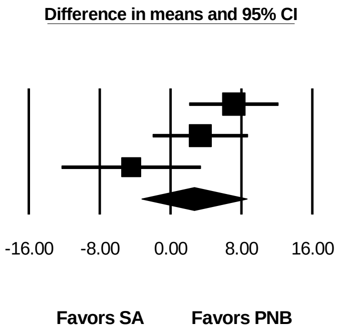

Supplementary Figure S31. **The trial sequential analysis for the studies comparing the effect of PNB to that of SA on the heart rate at T0**. Horizontal line represents the conventional boundaries for statistical significance. The blue solid line represents the cumulative z-curve. The number on the x-axis indicates required information size.

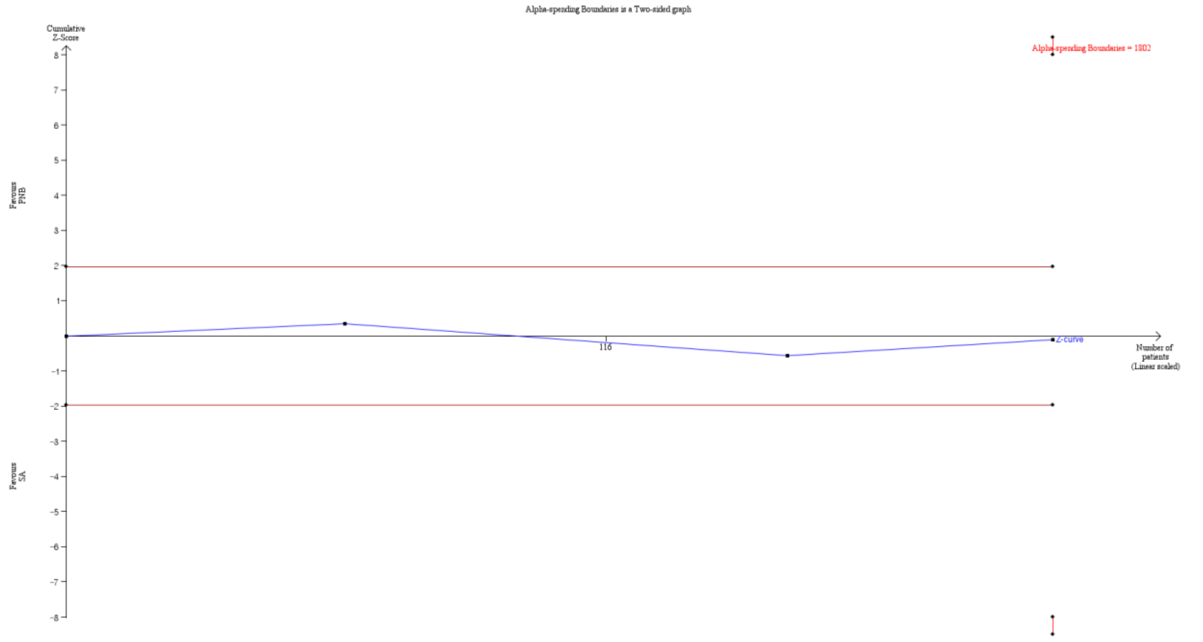

Supplementary Figure S32. The trial sequential analysis for the studies comparing the effect of PNB to that of SA on the heart rate at T30. Uppermost and lowermost curves represent trial sequential monitoring boundary lines for benefit and harm respectively. Horizontal line represents the conventional boundaries for statistical significance. Triangular lines on the right side reflects the futility boundaries. The blue solid line represents the cumulative z-curve. The number on the x-axis indicates required information size.

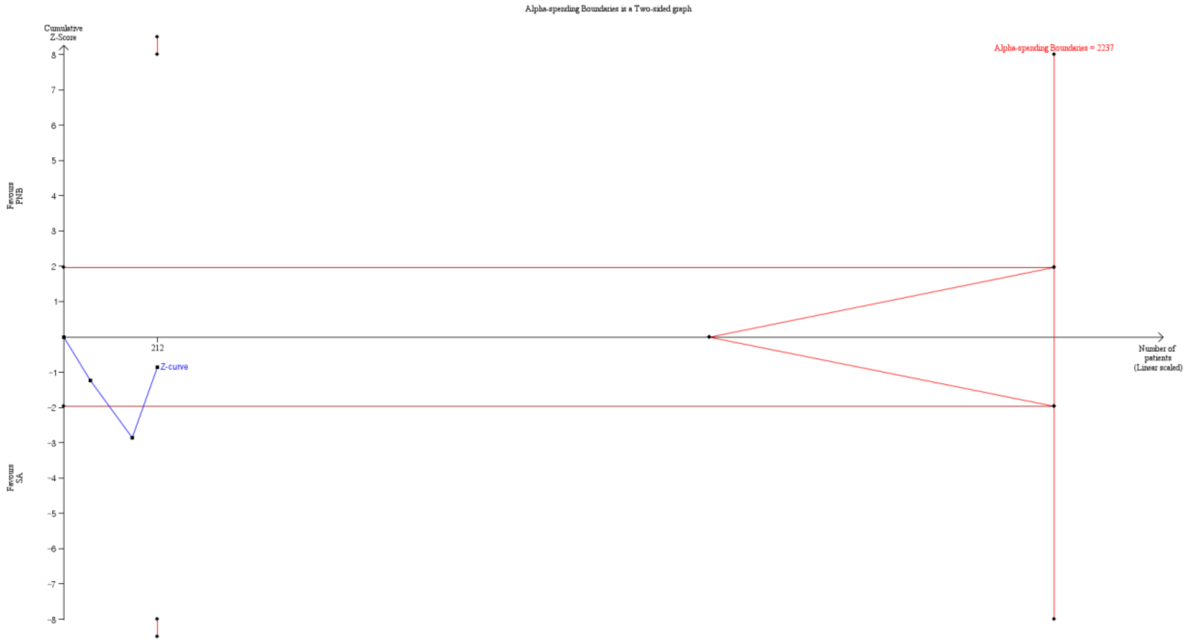

Supplementary Figure S33. **Forest plot for studies comparing the effect of PNB to that of SA on the urinary retention.** The figure depicts individual trials as filled squares with relative sample size and the 95% confidence interval (CI) of the difference as a solid line. The diamond shape indicates the pooled estimate and uncertainty for the combined effect.

Study name	Statistics for each study			Risk ratio and 95% CI
	Risk ratio	Lower limit	Upper limit	
Casati 2002	0.013	0.000	6.350	
Karaarslan 2016	0.050	0.000	28.081	
Protic 2010	1.056	0.000	6604.809	
Chauhan2023	1.111	0.000	7010.414	
	0.089	0.002	3.297	

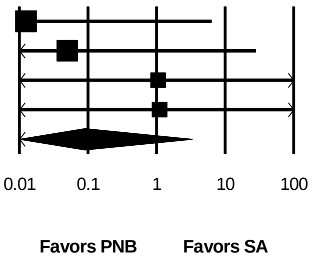

Supplementary Figure S34. The trial sequential analysis for the studies comparing the effect of PNB to that of SA on the urinary retention. Horizontal line represents the conventional boundaries for statistical significance. The blue solid line represents the cumulative z-curve. The number on the x-axis indicates required information size.

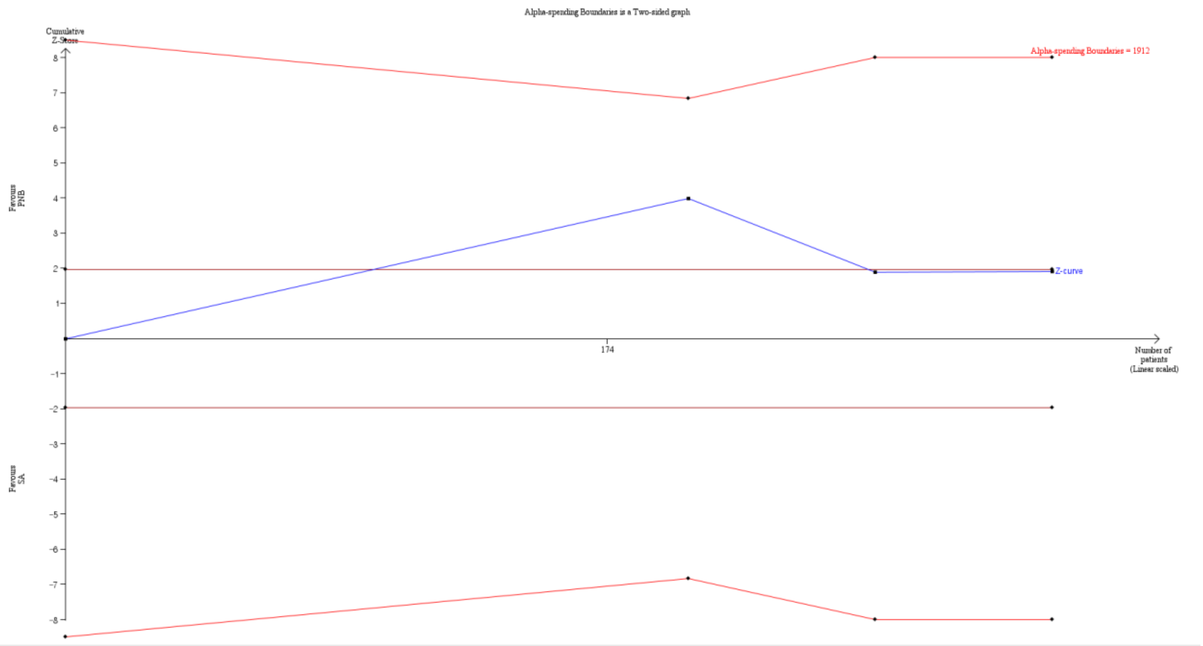

Supplementary Figure S35. Forest plot for studies comparing the effect of PNB to that of SA on the post-dural puncture headache. The figure depicts individual trials as filled squares with relative sample size and the 95% confidence interval (CI) of the difference as a solid line. The diamond shape indicates the pooled estimate and uncertainty for the combined effect.

Study name	Statistics for each study		
	Risk ratio	Lower limit	Upper limit
Karaarslan 2016	0.033	0.000	17.794
Protic 2010	0.106	0.000	68.027
Yang 2016	1.000	0.000	6337.642
Chauhan2023	1.111	0.000	7010.414
	0.159	0.004	6.071

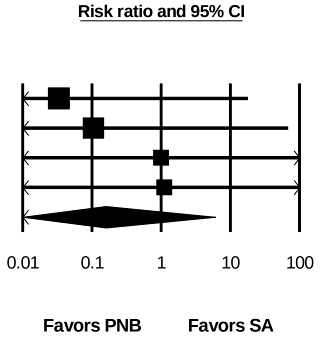

Supplementary Figure S36. The trial sequential analysis for the studies comparing the effect of PNB to that of SA on the post-dural puncture headache. Horizontal line represents the conventional boundaries for statistical significance. The blue solid line represents the cumulative z-curve. The number on the x-axis indicates required information size.

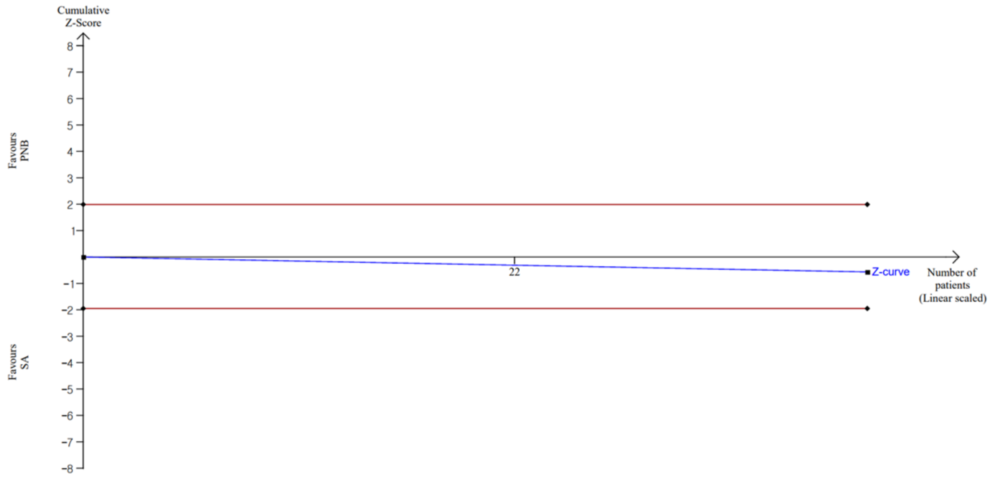

Supplementary Figure S37. Forest plot for studies comparing the effect of PNB to that of SA on the patient satisfaction. The figure depicts individual trials as filled squares with relative sample size and the 95% confidence interval (CI) of the difference as a solid line. The diamond shape indicates the pooled estimate and uncertainty for the combined effect.

Study name	Statistics for each study			Risk ratio and 95% CI
	Risk ratio	Lower limit	Upper limit	
Casati 2002	1.022	0.952	1.096	
Sort 2021	1.096	0.995	1.208	
	1.049	0.981	1.122	

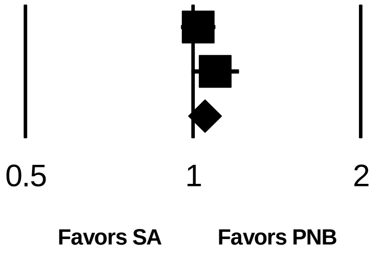

Supplementary Figure S38. The trial sequential analysis for the studies comparing the effect of PNB to that of SA on the patient satisfaction. Uppermost and lowermost curves represent trial sequential monitoring boundary lines for benefit and harm respectively. Horizontal line represents the conventional boundaries for statistical significance. Triangular lines on the right side reflects the futility boundaries. The blue solid line represents the cumulative z-curve. The number on the x-axis indicates required information size.

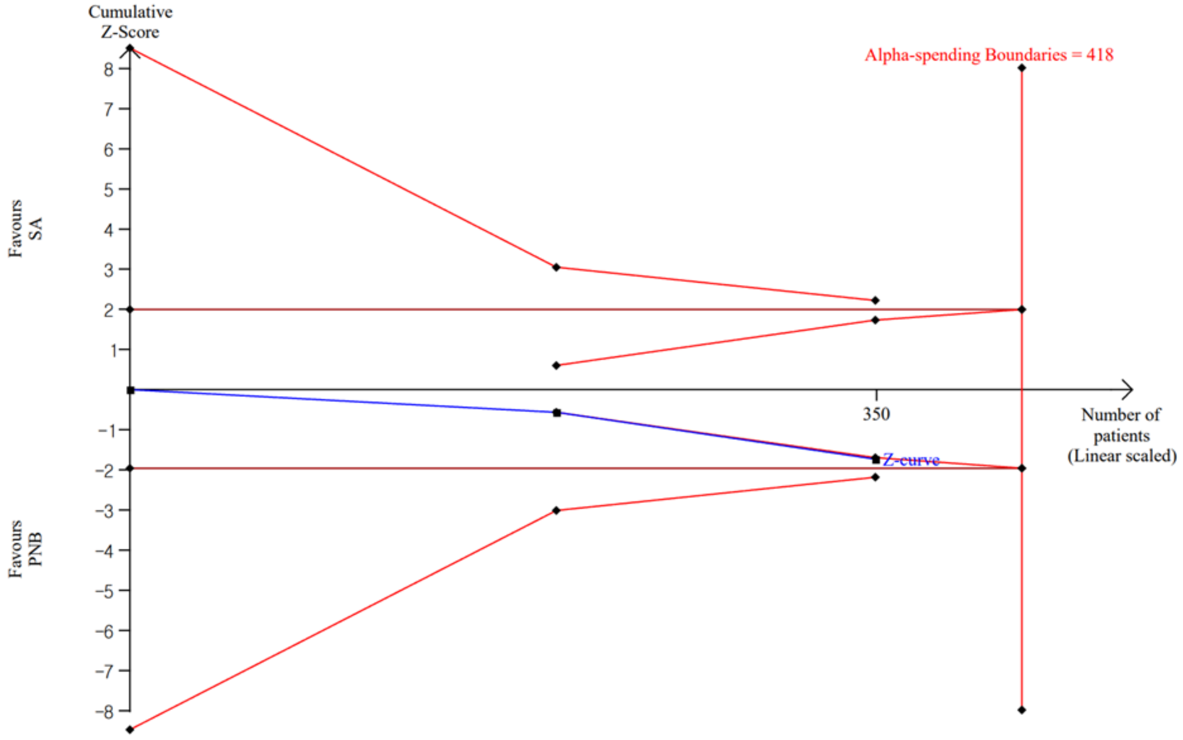

Supplement: Supplementary file 1 [file jpm-13-01096-s001.zip › jpm-2423287-supplementary.pdf]
